# Complete genomic sequence and analysis of β2 toxin gene mapping of *Clostridium perfringens* JXJA17 isolated from piglets in China

**DOI:** 10.1038/s41598-020-79333-8

**Published:** 2021-01-12

**Authors:** Xiu Zeng, Baosheng Liu, Jiao Zhou, Yimin Dai, Chunsheng Han, Linkang Wang, Yunzheng Wu, Jinhua Zhang

**Affiliations:** College of Animal Science and Technology, Jiangxi Agriculture University, Jiangxi, 330045 Nanchang China

**Keywords:** Genetics, Microbiology

## Abstract

*Clostridium perfringens* (Cp) is a ubiquitous opportunistic pathogen of humans and animals in the natural environment and animal intestines. The pathogenicity of Cp depends on the production of toxins encoded by genes on the chromosomes or plasmids. In contemporary literature, there is no clear consensus about the pathogenicity of CpA β2 toxin. To analyze the homology of the genome of piglet source CpA and its β2 toxin, we sequenced the whole genome of strain JXJA17 isolated from diarrhea piglets using the Illumina Miseq and Pacbio Sequel platforms. The genome was composed of a circular chromosome with 3,324,072 bp (G + C content: 28.51%) and nine plasmids. Genome and 16S rDNA homology analysis revealed a close relation of the JXJA17 strain with the JGS1495, Cp-06, Cp-16, and FORC_003 strains. These strains were isolated from different samples and belonged to different toxin-types. JXJA17 strain was found to carry two toxin genes (*plc* and *cpb2*). In contrast to other Cp strains, the *cpb2* of JXJA17 was located on a large plasmid (58 kb) with no co-localization of other toxin genes or antibiotic resistance genes. Analysis of JXJA17 genome homology and its *cpb2* would facilitate our further study the relationship between β2 toxin and piglet diarrhea.

## Introduction

*Clostridium perfringens* is a ubiquitous Gram-positive, rod-shaped anaerobic bacterium^1^. Based on the four secreted toxins (α, β, ε, and ι), this bacterium is categorized into five classical types (A to E); in addition, two other new types (F and G) were recently proposed based on the presence of *C. perfringens* enterotoxin (CPE) and necrotic enteritis toxin B (NetB)^2^. *C. perfringens* is a common pathogen in humans and animals and causes various diseases, including gas gangrene, diarrhea, and food poisoning^3^. The relationship between piglet diarrhea and toxins produced by *Clostridium perfringens* type A (CpA) is not well characterized^4,5^.

As an opportunistic pathogen, Cp produces many toxins and virulence factors closely related to the diseases it causes^6^. More than 18 toxins are produced by Cp types A-F. CpA predominantly produces α toxin and β2 toxin which is encoded by two *cpb2* alleles. β2 toxin is categorized into two types: consensus and atypical. The consensus *cpb2* is preferentially associated with *C. perfringens* isolated from pigs^7^. However, there is no clear consensus on the pathogenesis of CpA β2 toxin^3^. Farzan et al. found no significant difference in the presence of β2 toxin in the intestinal contents of normal and diarrheic piglets^8^. On the contrary, in 2003 Bueschel found that most porcine enteritis isolates were cpb2 positive (> 85%), while only 11.1% of normal porcine isolates contained cpb2^9^. There is a paucity of data related to the complete genome sequences of CpA isolated from diarrheal piglets. Moreover, there is an increasing interest in the toxin genes carried by the CpA plasmids for their impact on animal disease^10,11^.

In this study, strain JXJA17 was one of 229 Cp isolated from the rectal contents of neonatal piglets with diarrhea and was identified as CpA by toxin typing. This strain carries *cpb2*, which is more pathogenic than the CpA isolated from healthy piglets not carrying *cpb2*, based on animal experiments of mouse toxicity^12^ and rabbit ileal loop model. For better characterization β2 toxin of CpA isolated from piglet with diarrhea, we sequenced the JXJA17 complete genome and analyzed the *cpb2*.

## Results

### General features of JXJA17 genome

The sequencing coverage was 357 × with 5,250,394 high-quality reads, and 1,283,480,560 high-quality sequences (bp) were obtained on the Illumina MiSeq platform. The percentage of high-quality reads was 99.17%, reflecting the actual nucleotide composition. A total of 163,553 sequences were obtained on the PacBio Sequel platform. The total sequence length was 1,302,938,004 bp, and the GC content was 32.67%. The complete genome of JXJA17 was composed of a circular chromosome and nine extrachromosomal elements or plasmids. The genome had a chromosome with 3,324,072 bp, G + C content of 28.51%, 2967 ORFs (chromosome), 30 rRNA genes (10 5S, 10 16S, and 10 23S), and 95 tRNA genes. The identified extrachromosomal elements and their respective size (bp) were JXJA17_p1 (58,796), JXJA17_p2 (38,284), JXJA17_p3 (62,027), JXJA17_p4 (40,125), JXJA17_p5 (36,275), JXJA17_p6 (12,326), JXJA17_p7 (12,133), JXJA17_p8 (3,550), and JXJA17_p9 (11,801) (Table S1). rRNA genes, tRNA genes, or other non-coding RNA genes were not found in these plasmids, with the exception of JXJA17_p2 which had one tRNA gene and one non-coding RNA gene.

CRISPR is a unique family of DNA repeat sequences that are widely found in prokaryotic genomes. In the JXJA 17 genome, one CRISPR array was predicted. This array contained 2,865 bp (nucleotide positions 1,723,039 to 1,725,903), 44 repeats, and 43 spacer sequences. The length of each repeat was 36 bp, and the average length of the spacer sequences was 29 bp.

**Figure 1:**
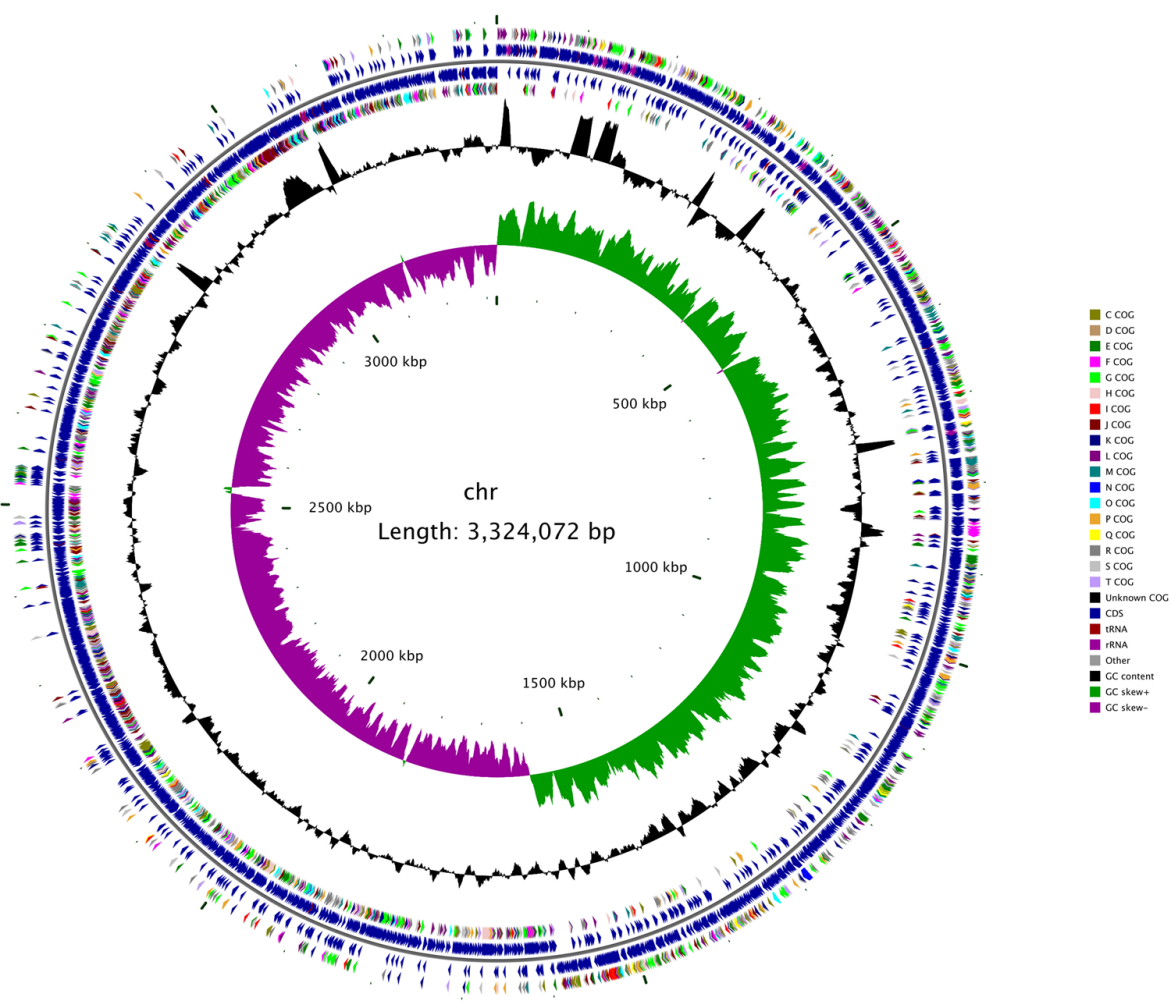
Circular map of the *Clostridium perfringens* JXJA17 strain chromosome. Summary of gene annotation and GC skew analysis of the genome of the JXJA17 strain. Circles (from inner to outer): circle 1 represents the scale; circle 2 shows the GC skew; circle 3 shows the GC content; circles 4 and 7 show the COG of each coding sequences (CDSs); and circles 5 and 6 show the positions of CDS. The genome atlas was drawn using CGView 1.0 (http://stothard.afns.ualberta.ca/cgview_server/).

Among the Clusters of Orthologous Groups (COG) categories in JXJA17, seven had the largest proportions (each with ≥ 5% of the total COG classifications): R (general function prediction only, 268 ORFs, 9.03%), G (carbohydrate transport and metabolism, 199 ORFs, 6.71%), S (function unknown, 192 ORFs, 6.47%), E (amino acid transport and metabolism, 180 ORFs, 6.07%), L (replication, recombination, and repair, 169 ORFs, 5.70%), J (translation, ribosomal structure, and biogenesis, 160 ORFs, 5.39%), and K (transcription, 160 ORFs, 5.39%). The detailed numbers of COG functional categories are shown in Fig. S1 and Table S2. The physical chromosome map with the location of functional genes, transcription in both directions from a specific site, and origins of replication (oriC) are shown in Fig. 1. The CDSs on the forward or reverse strand were identified by the GC skew value. The former was more inclined towards positive values, whereas the latter showed the opposite trend. The information that each CDS belonged to the specific COG category was analyzed.

### Homology analyses of JXJA17 genome

**Figure 2:**
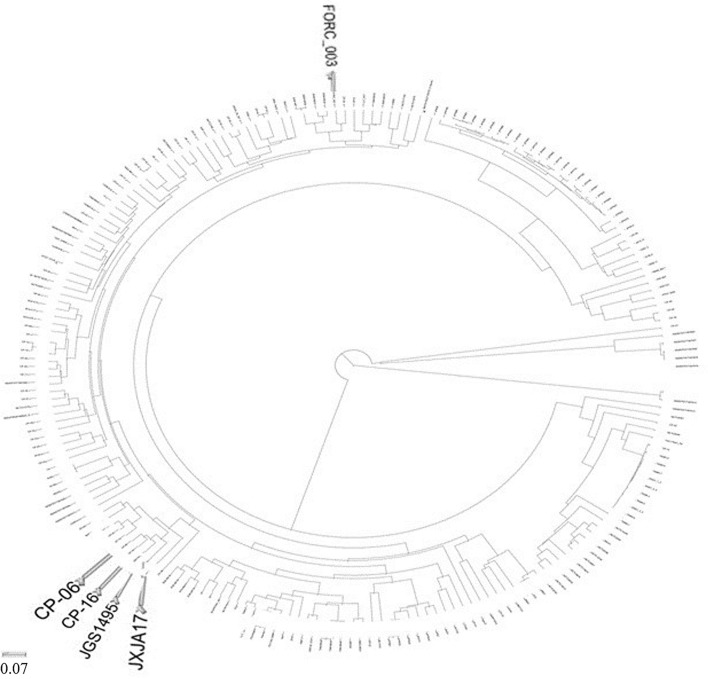
Phylogenetic tree depicting the relationship between JXJA17 and 205 reference Cp strains, constructed based on the genomic BLAST in NCBI (https://www.ncbi.nlm.nih.gov/genome/tree/158).

The phylogenetic tree based on genome between JXJA17 and all 205 Cp strains included in the NCBI genome database was constructed (Fig. 2). The dendrogram showed that the JXJA17 strain was more closely related to the JGS1495 (NZ_ABDU01000042.2, 84 contigs) strain and clustered with Cp-06(NZ_JAALNH010000001.1, 100 contigs) and Cp-16 (NZ_JAALMX010000001.1, 77 contigs) strains. However, the full genome sequences of these three *C. perfringens* were not de novo sequenced, and just spliced by some scaffolds and contigs. Therefore, the phylogenetic tree analysis is based on 16SrDNA genes and house-keeping genes between JXJA17 and 19 Cp strains complete genome using the i-sang platform (https://www.i-sanger.com/) ^13^ (Fig. S2 and S3). The dendrogram showed that JXJA17 strain was more closely related to the Cp FORC_003 (NZ_CP009557.1). Therefore, we compared and analyzed the general features of four *C. perfringens* genomes, and the results are shown in Table 1. Surprisingly, strain JXJA17 and its four genetically related strains were isolated from different hosts and environments, and their toxinotype were different as well. If a comparison were based on de novo sequencing, toxin genotyping of JXJA17 and Cp FORC_003 would be type A.

**Table 1:** General features of the *C. perfringens* genomes.

Strain/genome	C.*perfringens *JXJA17	C.*perfringens* CP-06	C.*perfringens* CP-16	C.*perfringens* JGS1495	C.*perfringerns* FORC-003
Genome size(bp)	3,324,072	3,534,593	3,407,131	3,661,329	3,395,109
Sequencing coverage	357x	300x	300x	N^a^	207x
Sequencing platform	Illumina MiSeq; PacBio	Illumina HiSeq	Illumina HiSeq	N^a^	PacBio
No. of contigs	1	100	77	84	2
Genes	3016	3289	3129	3359	3082
CDS()	2839	3126	2989	3101	2,910
tRNAs	95	63	65	94	97
N50 length(bp)	3,324,072	129,043	137,371	117,588	3,338,532
GC(%)	28.5	27.9	28	28.6	28.38
C.*perfringens* Symmetrical identity (%)^a^	92.1012	94.4809	94.4809	94.1446	93.7533
C.*perfringens* ATCC 13,124 Symmetrical identity (%)	86.7499	83.0388	84.8625	82.6052	88.4698
Host	Piglet	Pig	Deer	Pig	Aquarium water
Country	China	China	China	America	South Korea
Type^b^	A	C	A	C	A
ENA sample accession	SAMN08771210	SAMN14178512	SAMN14178522	SAMN02436294	SAMN03140316
ENA assembly accession	GCA_003350945.1	GCA_011063245.1	GCA_011063055.1	GCA_000171135.1	GCA_001304735.1

^a^This data is unknown.

^b^The strains are classified as A–E type according to α, β, ε and promethium toxins, excluding F and G types.

### Collinearity analyses of plasmid carrying cpb2 of JXJA17

**Figure 3:**
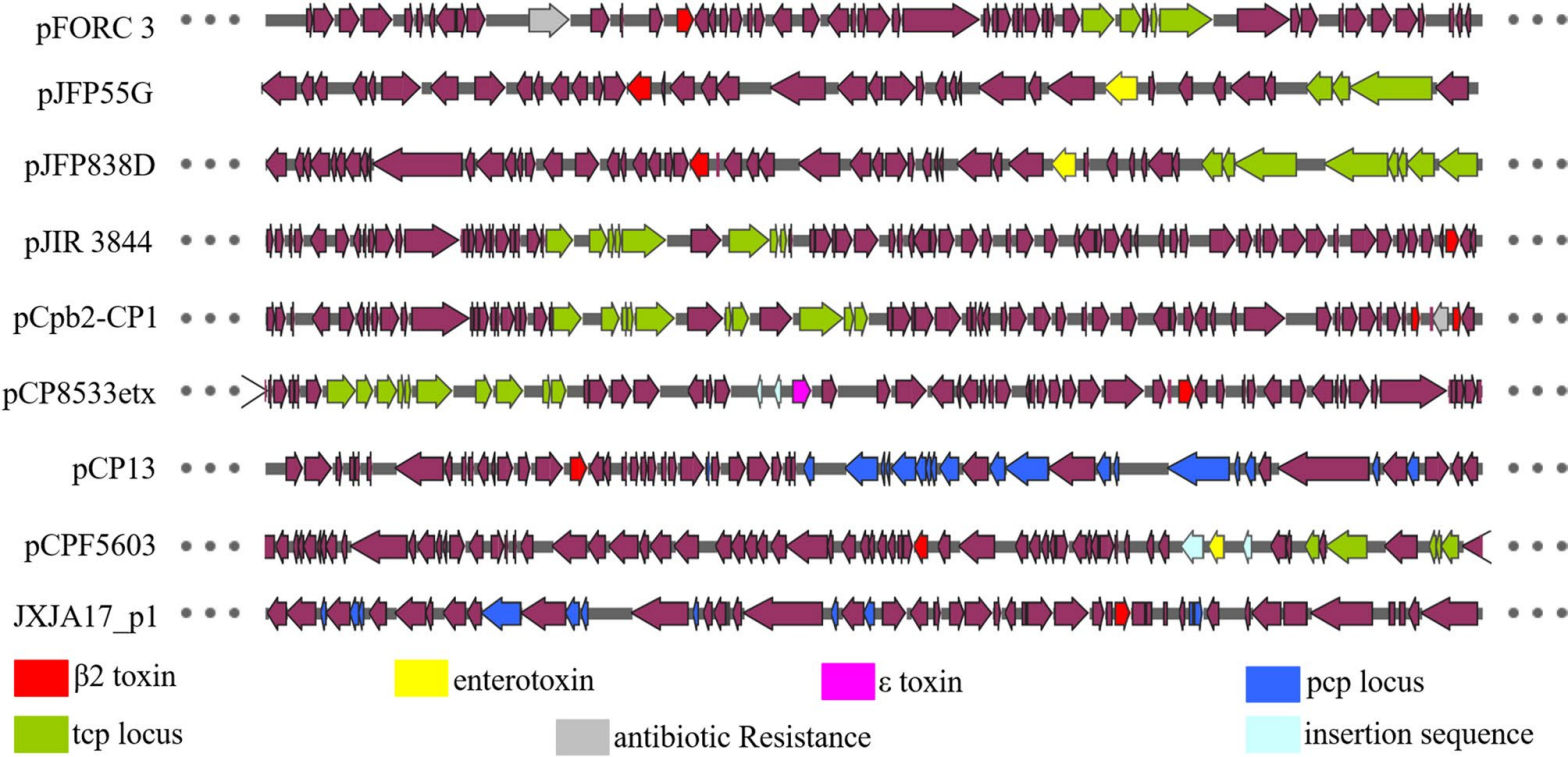
Comparative alignment of the sequenced *C. perfringens* plasmids carrying the *cpb*2. Arrows represent ORFs, and are colored as follows: red arrows—*cpb2*; yellow arrows—*cpe*; fuchsia arrows—*etx*; dark blue arrows—Pcp conjugation locus; green arrows—Tcp conjugation locus; gray arrows—antibiotic resistance; light blue arrows—insertion sequence. *ORFs* open reading frames.

**Figure 4:**
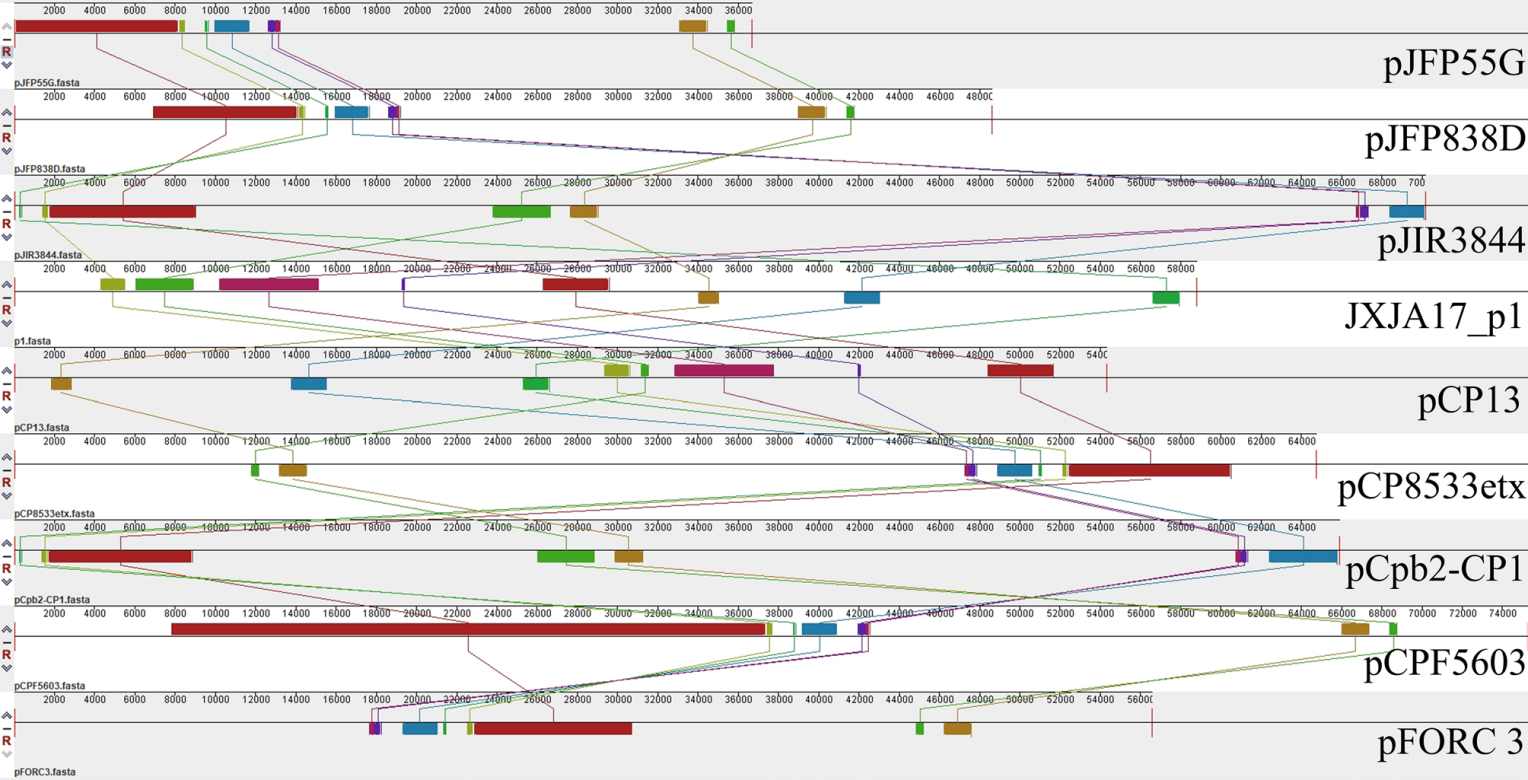
Collinearity analyses of plasmids JXJA17_p1, pFORC3, pJFP55G, pJFP838D, pJIR3844, pCpb2-CP1, pCP8533etx, pCP13, and pCPF5603. Results aligned by using Mauve 2.4.0. (http://darlinglab.org/mauve).

The sequencing results showed consensus *cpb2* (798 bp) in JXJA17. It (from 41,169 to 41,961 bp) was individually located on a large plasmid p1 (58,000 bp) and there was no co-carriage with other toxin (such as enterotoxin or ε toxin) genes and antibiotic resistance genes. In contrast to other plasmids carrying *cpb2*, no Tcp conjugation locus or insertion sequence (IS) was found in plasmid p1 of JXJA17 (Table 2 and Fig. 3). Collinearity analysis of plasmid carrying *cpb2* was conducted between JXJA17 and eight reference plasmids which were all *cpb2* encoding plasmids among the 50 Cp plasmids included in the NCBI. The results (Fig. 4) showed sharing of eight distinct locally collinear blocks (LCBs) by these plasmids, and *cpb2* was located in the blue LCB.

**Table 2:** General features of the plasmids that carry beta2 toxin in the Cp derived from NCBI database.

Plasmid	strain	Host	Size (bp)	CDS	GC%	Orther toxin	Tcp or Pcp	Antibiotic resistance	Insertion sequence
pFORC3	FORC_003	Human	56,577	56	27.28	–	Tcp	tetB(P)	–
pJFP55G	JP55	Horse	36,664	36	26.20	Enterotoxin	Tcp	–	–
pJFP838D	JP838	Canine	48,597	50	26.60	Enterotoxin	Tcp	–	–
pJIR3844	EHE-NE18	Chicken	69,606	68	25.55	–	Tcp	–	–
pCpb2-CP1	CP1	Chicken	65,875	73	26.79	–	Tcp	ErmB	–
pCP8533etx	NCTC8533B4D	N^a^	64,753	63	25.89	ε toxin	Tcp	–	IS231 IS1151
pCP13	13	Soil	54,310	53	25.5	–	Pcp	–	–
pCPF5603	F5603	Human	75,268	73	25.53	Enterotoxin	Tcp	–	IS1151 IS1469
JXJA17_p1	JXJA17	Piglets	58,796	58	25.67	–	Pcp	–	–

^a^This data is unknown.

### Pathogenesis and virulence factors

Some important pathogenic and virulence related genes were identified in the chromosome and plasmids of the JXJA17 strain. JXJA17 contains ten known virulence related genes, including the alpha toxin phospholipase C (PLC, CPE_RS00500) gene on the genome and the β2 toxin on JXJA17_p1. Other virulence factors identified in the genome were perfringolysin O (theta toxin, pfoA, CPE_RS01165), thiol-activated cytolysin (BAS3109), collagenase (kappa-toxin, colA, CPE_RS01210), hyaluronidase (mu-toxin, CPE_RS07880), exo-alpha-sialidase (CPE_RS04035), sialidase (nanH, CPF_RS07165), alpha-clostripain (cloSI, CPR_0833), and an ATP-dependent Clp protease proteolytic subunit (clpP, lmo2468) (Table S3). However, no other toxin-related gene was carried with *cpb2* in plasmid JXJA17_p1.

### Antibiotic resistance genes

Screening of the genome sequences against the Comprehensive Antibiotic Resistance Database (CARD) identified 21 antibiotic resistance genes and 18 antibiotic targets in the JXJA17 strain. This strain was most likely to develop resistance to fluoroquinolone, daptomycin, tetracycline, streptomycin, and erythromycin. The 21 antibiotic resistance genes (Table S4) included one aminocoumarin gene, three fluoroquinolone genes, four daptomycin genes, two tetracycline genes, one transmembrane protein gene, one dihydropteroate synthase gene, two elfamycin genes, one streptomycin aminoglycoside adenylyl transferase gene, one UDP-glucose-6-dehydrogenase gene, one Van response regulator gene, one two-component response regulator (*vanRF*) gene, and three unknown product genes. In addition, the gene encoding erythromycin resistance was found in the JXJA17_p4 plasmid. However, no antibiotic resistance gene was carried with *cpb2* in plasmid JXJA17_p1.

## Discussion

The genomic B LAST in NCBI and homology analysis of 16S rDNA revealed high homology of JXJA17 with CP-06, CP-16, JGS1495, and FORC_003 obtained from different hosts and geographical regions. The results indicated no significant correlation of the strains with the host sources, toxinotypes, or geographical distribution. Homology analysis based on 16S rDNA sequencing is a common method for *C. perfringens* taxonomy^14,15^. Among the 19 de novo sequenced *C. perfringens* included in the i-sang platform, the JXJA17 strain was most closely related to the Cp FORC_003 based on 16S rDNA homology analysis. The toxin genotyping of JXJA17 and Cp FORC_003 were type A, notwithstanding the fact that Cp FORC_003 was isolated from aquarium water in South Korea. Based on the above aspects, there is no significant correlation of the strains with the host sources, toxinotypes, or geographical distribution. The location of the pathogenic toxin on plasmids may be a key characteristic that facilitates the spread of *C. perfringens*.

The complete genome sequences of strain JXJA17 was found to contain nine extrachromosomal elements or plasmids. One of the plasmids contains the *cpb2*, and another plasmid carries the erythromycin resistance gene. Many plasmid-containing strains have recently been reported. Profeta et al. reported two plasmids in the *C. perfringens* netB positive strain 2016TE7641_69 isolated from the intestine of a diseased turkey in Italy^16^. In *C. perfringens* CBA7123 isolated from humans, Kim et al. identified a 46,640-bp plasmid (with a G + C content of 27.1%)^17^. Li. et al. identified four plasmids in *C. perfringens Del1* isolated from chicken^18^. The strain JP55 isolated from foal was found to contain five plasmids, and their G + C content was lower than that in the chromosome^19^. The G + C content of chromosomes from the above reported strains was higher than that in their respective plasmids. The JXJA17 strain had nine plasmids, it was also consistent with the rule that the G + C content of each plasmid is lower than that of its chromosome.

In this study, ten known virulence related genes were found in JXJA17. α toxin is the most toxic extracellular enzyme generated by *C. perfringens* type A^20^. This toxin is produced by all *C. perfringens* types and is essential for virulence. Furthermore, its core protein, phospholipase C, hydrolyzes phosphatidylcholine, and sphingomyelin, which are key constituents of eukaryotic cell membranes^21^. This toxin causes diseases such as myonecrosis^22^. The soluble toxins perfringolysin O and phospholipase C can cause host cell lysis and exhibit synergistic effects in *C. perfringens*-mediated gas gangrene^23^. β2 toxin is a very interesting compound. Some studies have indicated its lethal toxicity. Other reports indicate that β2 is an α auxiliary toxin^24,25^. Therefore, we are interested in the characteristics of β2 toxin from piglets CpA. In the JXJA17 strain, the size of plasmid carrying *cpb2* was 58 kb. This is consistent with previous studies in which the size of the plasmids encoding β2 toxin was 45–97 kb. Among the fifty plasmids of *C. perfringens* included in the NCBI, only eight plasmids contain *cpb2*, including one recombinant plasmid (pCpb-CP1)^26^. Of the seven natural plasmids, four carried *cpb2* along with another toxin (enterotoxin or ε toxin) and one plasmid was conjugated with antibiotic resistance gene. The plasmids pJFP55G and pJFP838D carrying the enterotoxin gene were from Cp JP55 and JP838 strains isolated from horses and dogs with necrotizing enteritis, respectively^19^. The plasmid pCPF5603 was from the Cp F5603 isolated from sporadic diarrhea (SPOR)^27^. The plasmid pCP8533etx carrying ε toxin was from CpB NCTC8533B4D. However, in JXJA17_p1, there were no other toxin genes, insertion sequence, antibiotic resistance gene, or Tcp conjugation locus conjugated with *cpb2*. By comparing these plasmids, JXJA17_p1 was most similar to plasmid pCP13, which was isolated from soil bacteria that cause gas gangrene. Plasmid pCP13 is a new family of conjugative toxin plasmids of C. perfringens strain 13^28^. CpCna, which encodes a putative collagen binding protein in the plasmid pCP13, is a potential virulence factor of porcine enteritis caused by *C. perfringens*^7^. The plasmid JXJA17_p1 contains the Pcp conjugation locus and CpCna. In addition, CRISPR is a prokaryotic immune system that recognizes foreign genes and silences their expression^29^. One CRISPR array predicted in the genome of JXJA 17 may lead to the sustained expression of toxin genes and their products. Further studies should investigate whether CRISPR in the genome would affect the expression of the toxin on JXJA17 plasmids.

In summary, we sequenced the complete genome and nine associated plasmids of the *C. perfringens* strain JXJA17. On 16SrDNA and whole-genome sequence analysis, the strain JXJA17 exhibited high homology with JGS1495, Cp-06, Cp-16, and Cp FORC_003. These strains were isolated from different hosts and environments, and their toxinotype were different as well. Public health authorities must be vigilant of the fact that *C. perfringens* carry multiple plasmids that harbor pathogenic toxin genes and are easily transmitted. Moreover, *cpb2* in JXJA17 was located on plasmid p1, and was independent of other toxins, antibiotic resistance genes, or insertion sequences. Plasmid JXJA17_p1 contains no Tcp but Pcp conjugation locus, which is a new family of conjugative toxin plasmids in *C. perfringens*^28^. Analysis of JXJA17 genome homology and its *cpb2* gene would facilitate our further study concerning the relationship between β2 toxin and piglet diarrhea.

## Methods

### Isolation and DNA extraction of *Clostridium perfringens* JXJA17

The study was approved by the Institution Animal Care and Use Committee of Jiangxi Agricultural University and performed according to its guidelines. All the piglets involved in the study were obtained after informed consent of the pig farm owner. We obtained the rectal contents by dipping rectal contents directly with sterilized cotton swabs through the anus of piglets. JXJA17 is a CpA strain isolated from the rectal contents of diarrhea piglets in the Jiangxi region of China and identified by biochemical tests, 16S rDNA sequencing, and toxin subtyping. A pure culture of the *C. perfringens* JXJA17 strain was obtained utilizing tryptose-sulfite-cycloserine agar (Haibo Biological Engineering Co. Ltd., Qingdao, China). Genomic DNA was extracted and sequenced by the Shanghai Personal Biotechnology Co. Ltd (Shanghai, China). The whole-genome sequence data was deposited in GenBank (Accession number CP028149).

### Whole-genome sequencing, assembly, and annotation

The genomic DNA was sequenced using the PacBio Sequel platform (SMRTbell Template Prep kit 1.0-SPv3, Pacific Biosciences, Menlo Park, CA, USA) and Illumina MiSeq platform (Rapid Plus DNA Lib Prep Kit, Illumina, USA). MiSeq reads were trimmed using AdapterRemoval 1.5.4 (https://adapterremoval.readthedocs.io/en/latest/) and SOAPec 2.0 (https://github.com/aquaskyline/SOAPdenovo2). Next-generation sequence data were assembled using the A5-MiSeq pipeline version 20160825, and contig and scaffold sequences were obtained. Data from the PacBio Sequel platform were assembled utilizing Canu 1.3 (https://canu.readthedocs.io/en/latest/), and scaffold sequences were identified. The locations and gaps among contigs from both sequencing results were fixed using MUMmer 3.2.3 (http://mummer.sourceforge.net/) ^30^. Assembly errors were corrected using Pilon version 1.22 (https://github.com/broadinstitute/pilon/releases/download/v1.22/pilon-1.22.jar), and the complete genome was obtained. The hypothesized coding DNA sequences (CDSs) were identified using Glimmer version 3.0 (http://cbcb.umd.edu/software/glimmer/), and the length of the open reading frame (ORF) was set to be no less than 110 bp. tRNA genes and ribosomal RNA genes were predicted using the tRNAscan-SE 2.0 (http://lowelab.ucsc.edu/tRNAscan-SE/index.html) and RNAmmer 1.2 Server (http://www.cbs.dtu.dk/services/RNAmmer/), respectively ^31^. Other non-coding RNA genes were obtained by comparing the genome sequences to the Rfam database (http://rfam.sanger.ac.uk). The clustered regularly interspaced short palindromic repeats (CRISPRs) recognition tool (CRT, http://crispr.u-psud.fr/crispr/) was used to predict CRISPR regions. Functional annotation of CDSs was achieved by searching a protein database and a non-redundant protein database of the National Center for Biotechnology Information (NCBI). The predicted proteins were functionally annotated using the evolutionary genealogy of genes: Non-supervised Orthologous Groups (eggNOG) database version 3 (http://eggnog.embl.de/version_3.0). Orthologs and paralogs were defined as the same protein when the degree of similarity of sequences was > 30%. The genome atlas was drawn using CGView 1.0 (http://stothard.afns.ualberta.ca/cgview_server/).

### Homology analysis

The complete genome sequence and annotation of 205 Cp strains were obtained from the GenBank database based on the whole-genome sequence of the JXJA17 strain. These strains were isolated from different hosts and the surroundings. To construct a dendrogram, we performed a homology analysis of 206 Cp in the NCBI database based on approximately 2967 genes of the JXJA17 using the genomic BLAST; the phylogenetic tree of 16S rRNA genes or house-keeping gene of 19 Cp which de novo sequencing for complete genome using the i-sang platform (https://www.i-sanger.com/). A list of the general features of the *C. perfringens* genomes which exhibit homology with JXJA17 was built.

### Comparative study of beta2 toxin-encoding plasmids

The key JXJA17-derived toxin genes are alpha and beta2. Alpha toxin gene, which is carried by *C. perfringens* types A-E, is known to be carried on genome; however, the *cpb2* is carried on plasmids. In order to explore the toxin genes of JXJA17, eight beta2 toxin-encoding plasmids were obtained from the GenBank using de novo assembly-graphs (https://www.ncbi.nlm.nih.gov/); subsequently, collinearity analysis was performed using the Mauve software. The sequences of eight plasmids were pFORC3 (NZ_CP009558.1/CP009558.1); pJFP55G (NZ_CP013042.1/CP013042.1); pJFP838D (NZ_CP013039.1/CP013039.1); pJIR3844 (NC_019257.1/JN689217.1); pCP8533etx (NC_011412.1/AB444205.1; pCpb2-CP1 (NC_019687.1/JQ655732.1); pCP13 (NC_003042.1/AP003515.1), and pCPF5603 (NC_007773.1/AB236337.1). The purpose of this analysis was to identify *cpb2* and align the plasmid 1 of JXJA17 with the eight reference beta2 toxin-encoding plasmids complete sequences.

### Prediction of virulence and antibiotic resistance genes

Potential virulence and antibiotic resistance genes in the complete genome were predicted using the virulence factors database (VFDB, http://www.mgc.ac.cn/VFs/main.htm) and the comprehensive antibiotic resistance database (CARD, http://arpcard.mcmaster.card) ^32^, respectively. Using BLAST (ftp://ftp.ncbi.nlm.nih.gov/blast/) to predict genes in the genome that are associated with virulence factors and antibiotic resistance, E-value threshold were set to 1e-6, more than 45% amino acid sequence consistency, and the ratio of the length of the sequence alignment to the length of the sequence not less than 70%.

## Supplementary Information

Supplementary Figures.

Supplementary Table S1.

Supplementary Table S2.

Supplementary Table S3.

Supplementary Table S4.
