# Supplementary material for: Complete genomic sequence and analysis of β2 toxin gene mapping of Clostridium perfringens JXJA17 isolated from piglets in China
Source: Sci Rep. 2021 Jan 12;11:475. doi: 10.1038/s41598-020-79333-8 (PMC7804025; doi:10.1038/s41598-020-79333-8)
Supplement: Supplementary file 1 — Supplementary Figures. [file 41598_2020_79333_MOESM1_ESM.docx]

**Supplementary Figure Legends**


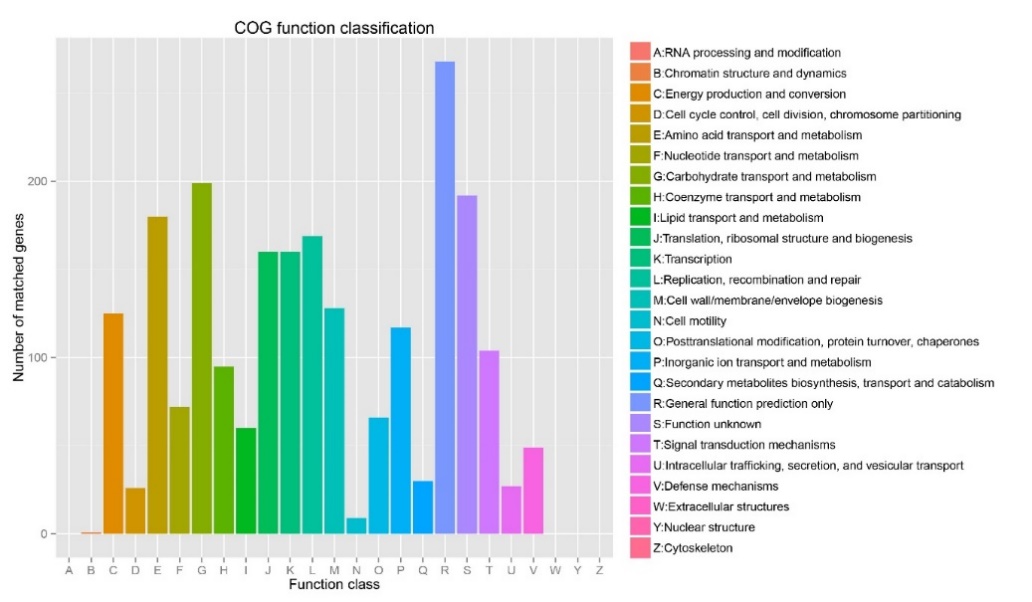


**Supplementary Figure S1.** The COG function annotation analysis diagram

COG, Clusters of Orthologous Groups


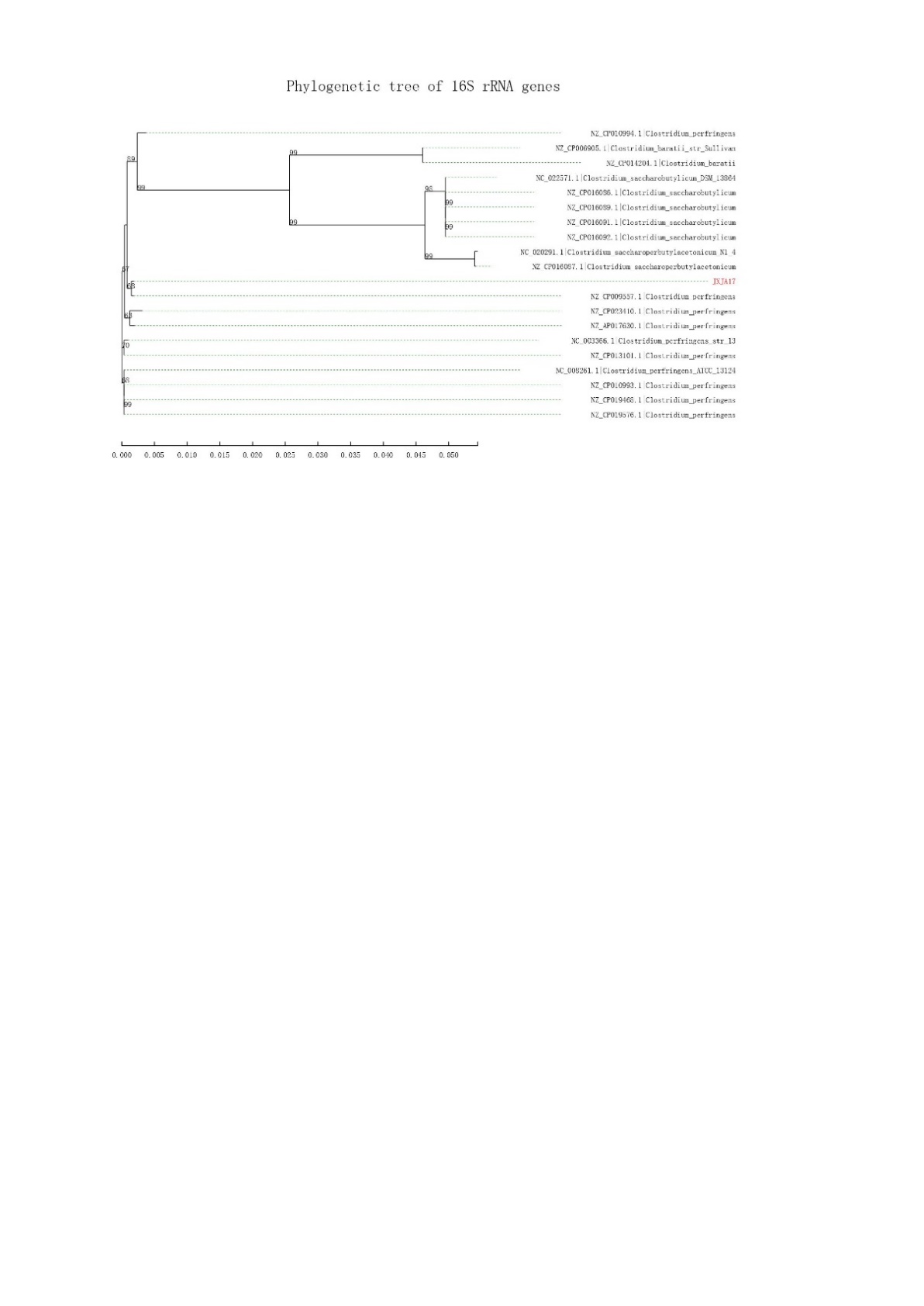


**Supplementary Figure S2.** Phylogenetic tree map based on 16S rRNA of *Clostridium perfringens*. By comparing with the local database of i-sang platform (<https://www.i-sanger.com/>), the 19 strains closest to the species level were selected based on 16s rRNA sequence, and the phylogenetic tree was constructed by NJ (Neighbor-Joining) method using MEGA 6.0 (<https://www.megasoftware.net/>).


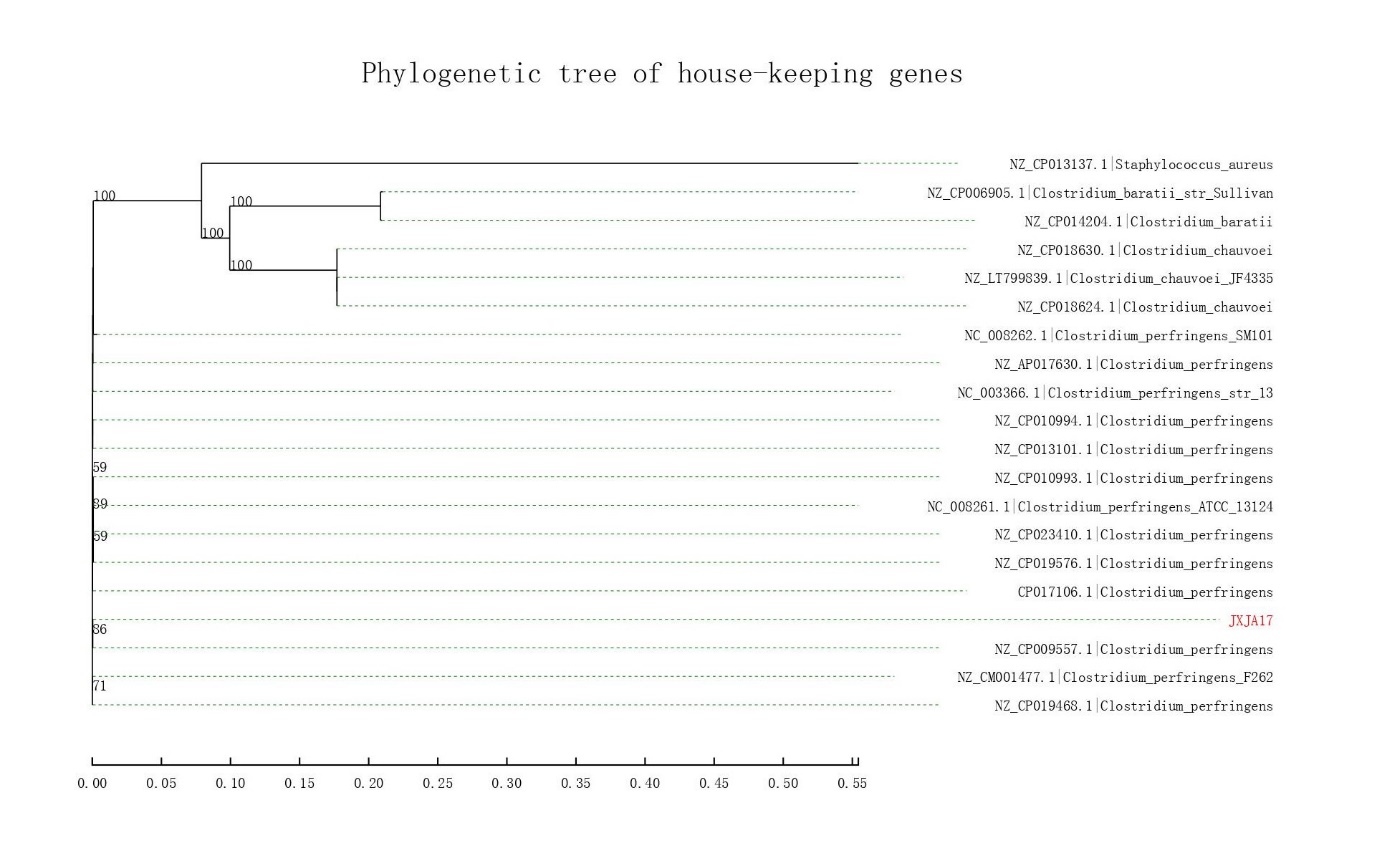


**Supplementary Figure S3.** Phylogenetic tree map based on 35 house-keeping genes. By comparing with the local database of i-sang platform (<https://www.i-sanger.com/>), the 19 strains closest to each other at species level were selected based on 31 housekeeping genes, and the phylogenetic tree was constructed by NJ (Neighbor-Joining) method using MEGA 6.0 (<https://www.megasoftware.net/>). The 31 house-keeping genes were *dnaG*, *frr*, *infC*, *nusA*, *pgk*, *pyrG*, *rplA*, *rplB*, *rplC*, *rplD*, *rplE*, *rplF*, *rplK*, *rpl*L, *rplM*, *rplN*, *rplP*, *rplS*, *rplT*, *rpmA*, *rpoB,* rp*sB*, *rpsC*, *rpsE*, *rpsI*, *rpsJ*, *rpsK*, *rpsM*, *rpsS*, *smpB and tsf*.
